# The N-terminal zinc finger domain of *Tgf2* transposase contributes to DNA binding and to transposition activity

**DOI:** 10.1038/srep27101

**Published:** 2016-06-02

**Authors:** Xia-Yun Jiang, Fei Hou, Xiao-Dan Shen, Xue-Di Du, Hai-Li Xu, Shu-Ming Zou

**Affiliations:** 1 College of Food Science and Technology, Shanghai Ocean University, Huchenghuan Road 999, Shanghai 201306, China; 2 Key Laboratory of Freshwater Aquatic Genetic Resources, Shanghai Ocean University, Huchenghuan Road 999, Shanghai 201306, China; 3 College of animal science and technology, Yangzhou University, Wenhui Road 48, Yangzhou 225009, China

## Abstract

Active *Hobo/Activator/Tam3 (hAT)* transposable elements are rarely found in vertebrates. Previously, goldfish *Tgf2* was found to be an autonomously active vertebrate transposon that is efficient at gene-transfer in teleost fish. However, little is known about *Tgf2* functional domains required for transposition. To explore this, we first predicted *in silico* a zinc finger domain in the N-terminus of full length *Tgf2* transposase (L-*Tgf2*TPase). Two truncated recombinant *Tgf2* transposases with deletions in the N-terminal zinc finger domain, S1- and S2-*Tgf2*TPase, were expressed in bacteria from goldfish cDNAs. Both truncated *Tgf2*TPases lost their DNA-binding ability *in vitro*, specifically at the ends of *Tgf2* transposon than native L-*Tgf2*TPase. Consequently, S1- and S2-*Tgf2*TPases mediated gene transfer in the zebrafish genome *in vivo* at a significantly (*p* < 0.01) lower efficiency (21%–25%), in comparison with L-*Tgf2*TPase (56% efficiency). Compared to L-*Tgf2*TPase, truncated *Tgf2*TPases catalyzed imprecise excisions with partial deletion of TE ends and/or plasmid backbone insertion/deletion. The gene integration into the zebrafish genome mediated by truncated *Tgf2*TPases was imperfect, creating incomplete 8-bp target site duplications at the insertion sites. These results indicate that the zinc finger domain in *Tgf2* transposase is involved in binding to *Tgf2* terminal sequences, and loss of those domains has effects on TE transposition.

Transposable elements (TEs) are discrete DNA segments that are able to move from one locus to another within genomes of host cells using a cut-and-paste mechanism^1^,^2^. Their wide distribution among all major branches of life, their diversity, and their intrinsic biological features have made TEs a considerable source of genetic innovations during species evolution^3^,^4^. Moreover, transposons may be valuable genomic tools for transgenesis, insertional mutagenesis and DNA delivery vehicles in gene therapy^5^,^6^,^7^,^8^,^9^. In eukaryotic genomes, DNA transposons have been classified into approximately 20 superfamilies based on amino acid sequence similarities of their encoded transposases^10^,^11^.

The *hAT* superfamily of transposons, named after the *Drosophila* element *hobo*, McClintock’s maize *Activator* and snapdragon *Tam3*, is widespread in plants and animals^12^. All *hAT* elements share several defining features, including terminal inverted repeats (TIRs) and subterminal repeats (STRs) at each end of the TE, and a gene encoding ~600–800 amino acid transposase that catalyzes DNA cleavage and target integration, with 8 bp target site duplications (TSDs) at both ends of the integration site during transposition^13^,^14^,^15^. In vertebrates, most *hAT* transposons are inactive, as host cells have developed the mechanism of vertical inactivation to silence and avoid the deleterious effects of active transposons on genome stability^16^. Thus, only a few active vertebrate elements have been discovered. *Tol2*, the first autonomous vertebrate *hAT* transposon, was identified in medaka (*Oryzias latipes*) and has proven active in a variety of vertebrate cell types^17^,^18^. The goldfish (*Carassius auratus*) *Tgf2* transposon is another autonomously active vertebrate *hAT* transposon^19^,^20^. The *Tgf2* element is 4,720 bp long^21^, and the full length *Tgf2* transposase is 686 aa long; variant isoforms naturally occur in the goldfish due to the different starting positions of the coding frame^19^. Although it is capable of mediating gene transfer effectively in different teleost fish^19^,^20^,^21^,^22^, the functional domains of the *Tgf2* transposase are poorly understood at the mechanistic level. The exploration of its role in the transposition process is crucial to understanding its mechanisms for catalyzing excision and transposition.

In this study, the domain architecture of *Tgf2* transposase was predicted based on *in silico* analysis of its amino acid sequence. Two cDNAs were cloned from goldfish embryos, which encoded two truncated *Tgf2* transposases with deletions of the N-terminal zinc finger domain. The biological functions of prokaryotically expressed truncated *Tgf2*TPases were assessed by *in vitro* DNA binding assay and *in vivo* transposition activity in zebrafish model. Our results show that the zinc finger domain in *Tgf2* transposase is involved in binding to *Tgf2* terminal sequences, and mutations to this domain have effects on the transgenic efficiency and integration patterns during the transposition process. Our work may facilitate the development of improved genomic tools, and provide insight into aspects of the transposition process of *Tgf2* element.

## Materials and Methods

### Experimental animals

Ryukin goldfish (*Carassius auratus*) embryos were provided by the Jujin ornamental fish farm, Shanghai, China. The wild-type Tübingen strain of zebrafish (*Danio rerio*) maintained in our laboratory was used for mating, spawning, microinjection, and transposition activity analysis in this study. All animal experiments were performed in accordance with the Shanghai Ocean University Committee on the Use and Care of Animals and were approved by the Committee on the Use and Care of Animals at Shanghai Ocean University.

### Sequence analysis and modeling of full-length Tgf2 transposase

Functional domains of the goldfish full-length *Tgf2* transposase (L-*Tgf2*TPase, 686 amino acids) were predicted using Phyre2 (http://www.sbg.bio.ic.ac.uk/phyre2/)^23^. A three-dimensional model of the L-*Tgf2*TPase monomer was generated using Phyre2 and protein structures were visualized using PyMol (www.pymol.org), based on a homology model of *Hermes* transposase protein^24^. Nuclear localization of L-*Tgf2*TPase was predicted on cNLS mapper (http://nls-mapper.iab.keio.ac.jp)^25^. Alignment of conserved amino acid sequences of DDE-based catalytic domains of *hAT* transposases from different species was performed with the Clustal X 1.81 program^26^.

### Plasmid construction, prokaryotic expression and purification

Three cDNAs (2061bp, 1734 bp and 1692 bp) encoding goldfish wild type *Tgf2* transposases of L- (1–686 aa.), S1- (110–686 aa.) and S2-*Tgf2*TPase (124–686 aa.) were previously isolated from Ryukin goldfish embryos^19^. In addition to the 5′-truncated region, cDNAs of both S1- and S2-*Tgf2*TPases were identical to those in L-*Tgf2*TPase. The recombinant vector pET-28a(+)-L-*Tgf2*TPase was constructed for L-*Tgf2*TPase, and was prokaryotically expressed and purified in our previous publication^27^. The control plasmid pET-28a(+)-L-*Tgf2*TPase^D228N, E648Q^, mutated in the catalytic domain of *Tgf2* transposon, was constructed on the basis of the plasmid pET-28a(+)-L-*Tgf2*TPase, using the QuikChange Lightning multi site-directed mutagenesis kit from Stratagene. The primer set for aspartic acid (D)-to-asparagine (N) mutation at position 228 was 5′-GCAACCACAACGAATTGTTGGACTGCACGTAGAAAGTCATTC-3′ and 5′-CCAACAATTCGTTGTGGTTGCAATCCATTCAACTTCACTCAT-3′. The primer set for glutamic acid (E)-to-glutamine (Q) mutation at position 648 was 5′- GGCTGCCTGTCAGAGGCTTTTCAGCACTGCAGGATTGCTTTT -3′ and 5′- AAAAGCCTCTGACAGGCAGCCGATGCGGGAAGAGGTGTATTA -3′. All reactions were performed according to the manufacturers specifications, and positive clones were examined by PCR and direct sequencing. Using S1-*Tgf2*TPase cDNA as template, the coding region for S1-*Tgf2*TPase was amplified by PCR using *Pfu* DNA polymerase (Stratagene, La Jolla, CA, USA) using the primer set 5′-CGCGGATCCATGAAACGTAAAATTGGCA-3′ and 5′-CCGCTCGAGTTATTCAAAGTTATAAAAACG-3′. Using S2-*Tgf2*TPase cDNA as template, the coding region for S2-*Tgf2*TPase was amplified by the primer set 5′-CGCGGATCCATGCATCCGAACTATCT-3′ and 5′-CCGCTCGAGTTATTCAAAGTTATAAAAACG-3′. These coding regions were both cloned into pMD-19T vector (Takara, Dalian, China). For expression of the truncated recombinant *Tgf2*TPases in *E. coli*, the coding regions for S1- and S2-*Tgf2*TPase were subcloned into the pET-28a(+) vector (Merck, Shanghai, China).

Recombinant vectors were then used to transform *Rosetta1 (DE3)* competent cells (Merck, Shanghai, China). *E. coli* cells containing recombinant plasmid harboring S1- or S2-*Tgf2*TPase were initially induced at the early log phase of culture (OD600 = 0.3–0.4) with 0.8 mM IPTG for 6 h at a low-temperature (22 °C) as previously described^27^. Recombinant proteins were purified with a Ni^2+^-affinity column in a FPLC AKTA Purifier system (GE Healthcare, Piscataway, USA). Recombinant TPases were identified using ABI 4800 Plus MALDI TOF/TOF™ (Applied Biosystems, Foster City, USA) and MS/MS ion searches using Mascot (Matrix Science Ltd, London, UK).

### DNA-binding activity assay

The DNA-binding activities of truncated recombinant S1-*Tgf2*TPase, S2-*Tgf2*TPase and L-*Tgf2*TPase^D228N,E648Q^, and previously purified full-length L-*Tgf2*TPase^27^ were evaluated by size exclusion chromatography methods used for other transposons^13^,^24^,^28^,^29^. Since transposases are proposed to bind transposon terminal regions^30^,^31^, a 50 bp DNA probe containing TIR and STR sequences was designed for a binding assay based on at the left end of *Tgf2* (GenBank Accession No. HM146132, 4720bp), named as L50 (5′- CAGAGGTGTAAAAGTACTTAAGTAATTTTACTTGATTACTGTACTTAAGT-3′). Oligonucleotides were synthesized and PAGE-purified (Sangon Ltd, Shanghai China), and annealed with their complementary motifs. DNA-binding activity was determined as previously described^13^,^24^,^28^,^29^, with certain modifications. Briefly, double stranded DNAs were mixed with 20 mM *Tgf2*TPase at a 1:1 molar ratio of protein: DNA in a buffer containing 0.5 M NaCl. The mixture was then dialyzed into buffer (pH 7.5) containing 20 mM Tris (pH 7.5), 0.2 M NaCl and 5 mM DTT overnight. Dialyzed fluids (50 μL) were applied to a Superdex 200 column (GE Healthcare, Piscataway, USA) equilibrated with the same buffer (pH 7.5) and then eluted at 4 °C at a rate of 50 μL/min. DNA-binding activity was assessed by the formation of stable complexes under size exclusion chromatography conditions. A random 50-mer double-stranded DNA C50 (5′- CCTGTACTCACGGCATTGCCATTGGCTCGTCTACGCTAGCTCCGCGCTGA-3′) was used as a control.

### Microinjection

The donor plasmid of pTgf2-EF1α-EGFP, containing 220 bp and 185 bp of the left and right ends of the goldfish *Tgf2* transposon and driven by the *Xenopus EF1a* promoter was constructed as described previously^19^. A mixture of 50 pg donor plasmid (pTgf2-EF1α-EGFP) was injected alone, or with 50 pg recombinant S1-, S2- or L-*Tgf2*TPase into fertilized zebrafish eggs at the one- to two-cell stage (~1 nl/embryo). After injection, embryos were placed in embryo rearing medium and maintained at room temperature. EGFP fluorescence in embryos was analyzed at 12, 24 and 96 hours post fertilization (hpf) using a Nikon SMZ 1500 fluorescence microscope.

### Transposition rate and insertion site analysis

Total genomic DNA was isolated from zebrafish tail fin clips (0.1 to 0.2 g,^32^). The primers for PCR analysis of transposition or integration rate of EGFP were: EGFP-f, 5′-ACCCTCGTGACCACCCTGAC-3′; EGFP-r, 5′-GCTTCTCGTTGGGGTCTTTGCTC-3′. PCR, cloning and sequencing were conducted as previously described^19^,^27^.

The flanking sequences of the transposon insertion sites were analyzed using the GenomeWalker^TM^ Universal Kit (Clontech, California, USA), as previously described^19^,^27^. For splinkerette PCR, 25 μg genomic DNA was digested for 12–16 h at 37 °C with 80 U of *Stu* I and *EcoR* V in a 100 μl reaction volume, was purified by ethanol precipitation, and then 4 μl of the digestion mix was ligated with the splinkerette adaptor overnight at 16 °C. The linker ligation was used as a template for two rounds of PCR to amplify the transposon/genome junction. The nested primers for the 5′ flanking sequences were 5′-AACAGCTCCTCGCCCTTGCTCACCAT-3′ and 5′-ACCGTCGCTGGCTTTTGTGTTACACG-3′. The nested primers for the 3′ flanking sequences were 5′- TCGGCATGGACGAGCTGTACAAGTAA-3′ and 5′-CCTCTACAAATGTGGTATGGCTGATTA-3′. The amplified fragments were cloned into the pMD19-T vector (TaKaRa, Dalian, China), transformed into *DH5α E. coli* cells and positive clones were examined by PCR and direct sequencing.

### Statistics

Values are expressed as mean ± S.E. Differences among groups were analyzed by one-way ANOVA followed by Fisher’s Post Hoc tests or unpaired *t*-test. Significance was defined as *p* < 0.01.

## Results

### Molecular architecture of the goldfish full-length Tgf2 transposase

**Figure 1:**
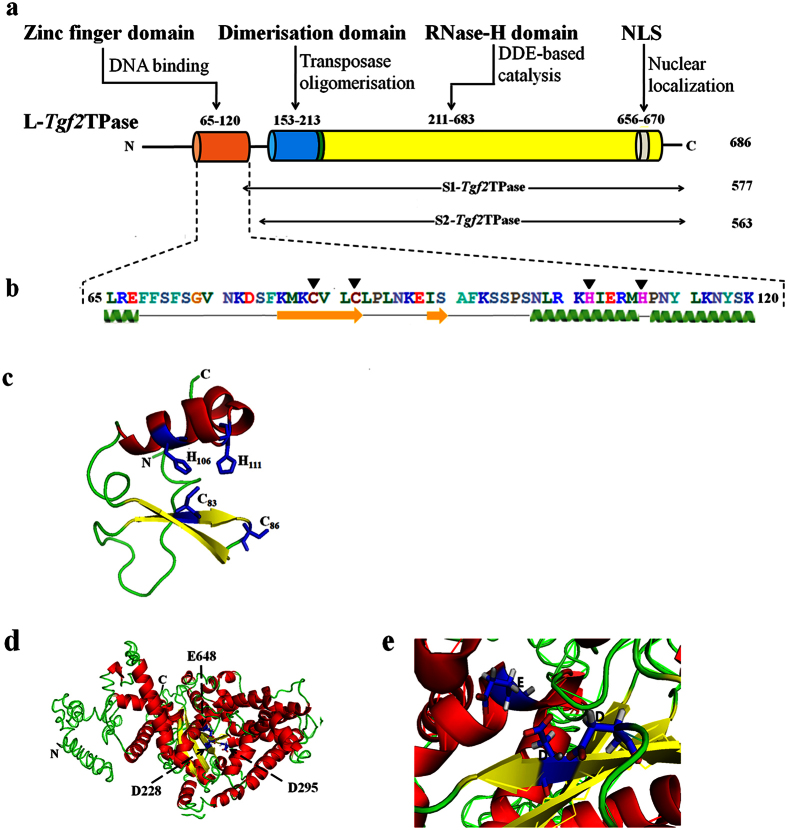
Molecular architecture of *Tgf2* transposase. (**a**) The domains of full length *Tgf2* transposase (L-*Tgf2*TPase) (686 aa). (**b**) The secondary structure of the N-terminal zinc finger domain. Two conserved cysteine (C) and two conserved histidine (H) residues are indicated with dark triangles. The α-helices (green) and β-strands (yellow) are also shown. (**c**) The three-dimensional model of the N-terminal zinc finger domain, which consists of an α-helix (red), two antiparallel β-sheets (yellow), and a motif usually used for DNA-binding (green). Two histidine (H_106_, H_111_) and two cysteine (C_83_, C_86_) residues (blue) form a cave that can bind a zinc ion. (**d**) The homology model of the full length *Tgf2* transposase. α-helices and β-strands are colored in red and yellow respectively. Residues of the DDE (D_228_, D_295_ and E_648_) active site triads are labeled. (**e**) Close-up view of the DDE active site.

Amino acid sequence analysis on Phyre2 suggested that the full length *Tgf2* transposase (L-*Tgf2*TPase) consisted of four functional domains (Fig. 1a). First is an N-terminal BED zinc finger domain (Cx_2_Cx_19_Hx_4_H, 65–120 aa) involved in DNA binding, two zinc binding residues (C_83_ and C_86_) comprise a zinc knuckle at the end of the β-hairpin, and two other residues (H_106_ and H_111_) are at the C-terminal end of the α-helix, which is composed of a β-hairpin followed by an α-helix that forms a left-handed ββα unit (Fig. 1b,c). The second presumptive domain identified is a dimerization domain defined by amino acids 153–213 (Fig. 1a), presumably involved in the formation of oligomers, as well as in DNA binding. A C-terminus RNase-H domain comprises amino acids 211 to 683 (Fig. 1a). This is presumably the core catalytic domain for DNA excision and transposition^15^,^33^,^34^. A 3D model of *Tgf2* transposase (Fig. 1d) was constructed with 95% accuracy based on the housefly hAT *Hermes* transposase, the only recently available crystal structure for a *hAT* transposase^24^.

**Figure 2:**
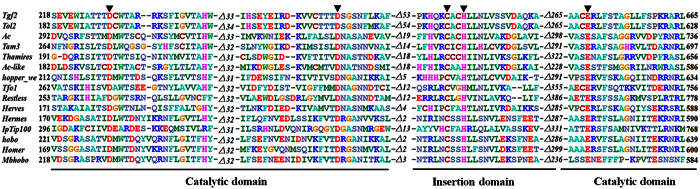
Alignment of conserved amino acid sequences of the C-terminus RNase-H domain of 15 *hAT* transposases. Distances between the conserved blocks are indicated by the number of amino acid residues. Conserved DDE residues and CX2H motif are marked with dark arrows. Accession numbers: *Tgf2 (Carassius auratus*): AFC96942; *Tol2 (Oryzias latipes*): BAA87039; *Ac (Zea mays*): CAA29005; *Tam3 (Antirrhinum majus*): CAA38906; *Thamires (Saintpaulia hybrid cultivar*): BAJ23914; *Ac-like (Amaranthus palmeri*): AGH20188; *hopper_we (Bactrocera dorsalis*): AAL93203; *Tfo1 (Fusarium oxysporum*): BAA32244; *Restless (Tolypocladium inflatum*): CAA93759; *Herves (Anopheles gambiae*): AAS21248; *Hermes (Musca domestica*): AAC37217; *IpTip100 (Ipomoea purpurea*): BAA36225; *hobo (Drosophila melanogaster*): AAA51465; *Homer (Bactrocera tryoni*): AAD03082; *Mbhobo (Mamestra brassicae*): AAA51465.

**Figure 3:**
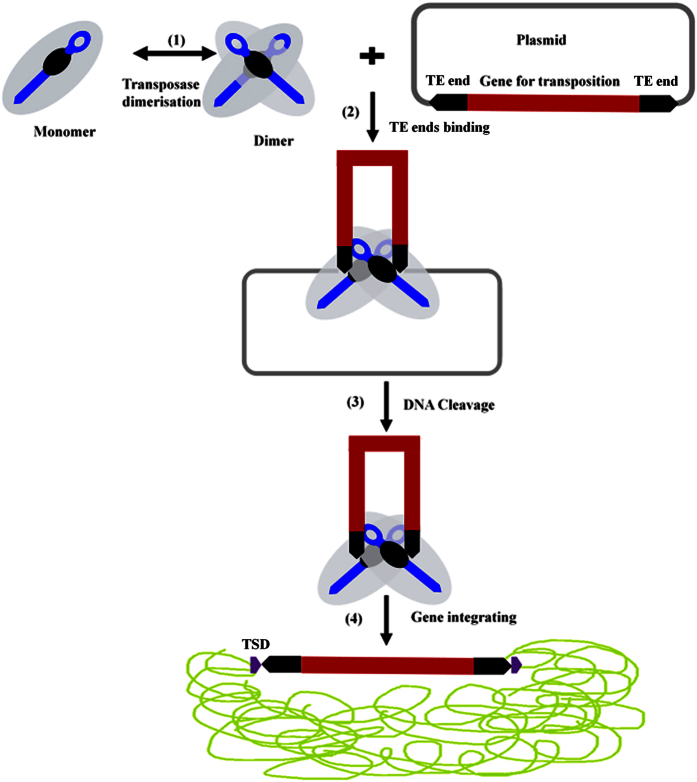
Proposed model for *Tgf2* transposition. The cut-and-paste process contains four main stages: (1) pairing of transposases by dimerization or oligomerization, (2) DNA-specific binding of the transposases to the TE ends flanking the transposon, (3) cleavage of DNA strands at the ITR ends of the transposon, and (4) capture of a new locus and integration of target DNA, purple block arrows indicated target site duplications (TSDs) in the host cell.

Using sequence alignment with other *hAT* transposases^10^,^12^,^15^,^17^,^34^, three conserved amino acids residues (DDE) were identified in the RNase-H catalytic domain of *Tgf2* transposase (Fig. 2). Residues of the DDE (D_228_, D_295_ and E_648_) are extremely close in their spatial distribution (Fig. 1e). A CX_2_H motif within the RNase-H catalytic domain of *Tgf2* transposase is also identified (Fig. 2), which functions as insertion domain for the correct positioning of the final E648 residue of the catalytic triad in the active site^24^,^34^. These conserved residues or motifs were exploited as phylogenetic characteristics to infer evolutionary relationships among *hAT* transposases, indicating their importance for the functioning of the enzyme^15^,^34^,^35^. Finally, a monopartite nuclear localization signal (NLS, 656–670 aa.) was found at the C-terminus (Fig. 1a). Based on the domain organization of the *Tgf2* transposase as described above, and data on regulation from other class II transposable elements^28^,^31^,^36^, a *Tgf2* transposition model was proposed (Fig. 3).

### N-terminal truncated Tgf2 transposases lose their DNA-binding activity

**Figure 4:**
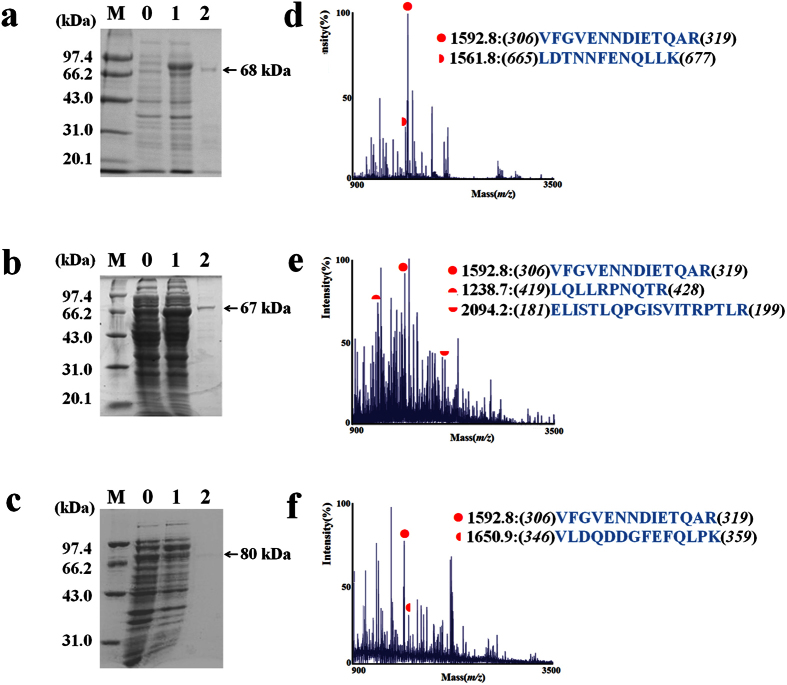
Purification and identification of recombinant *Tgf2*TPases. (**a–c**) SDS-PAGE analysis of S1-*Tgf2*TPase (**a**) and S2-*Tgf2*TPase (**b**) with N-terminal deletions, or of mutated L-*Tgf2*TPase^D228N, E648Q^ (**c**) expression from *E. coli* strain *Rosetta 1* (DE3) by vector pET-28a(+). M, standard molecular marker. 0, without IPTG. 1, with IPTG. 2, purification from eluted peak of Ni^2+^-affinity column. (**d–f**) Mass spectrometry of S1-*Tgf2*TPase (**d**), S2-*Tgf2*TPase (**e**) or L-*Tgf2*TPase^D228N, E648Q^ (**f**) collected from eluted peak of Ni^2+^-affinity column. The confirmed fragments of *Tgf2*TPase are indicated in red, with the corresponding peptide sequences listed.

To investigate the function of the N-terminal zinc finger domain in DNA binding, two truncated *Tgf2* transposase proteins, designated S1- and S2-*Tgf2*TPase, were prokaryotically synthesized in *E. coli*. The S1-*Tgf*2TPase was characterized by deletion of the N-terminal zinc finger domain, while S2-*Tgf2*TPase included an additional deletion of part of the linker region between the zinc finger domain and the dimerization domain (Fig. 1a). The double mutation L-*Tgf2*TPase^D228N, E648Q^, with an intact N-terminal zinc finger domain, was used as control. The recombinant S1- (~68 kDa), S2- (~67 kDa) and L-*Tgf2*TPase^D228N, E648Q^ (~80 kDa) proteins were successfully expressed in a soluble form, purified with Ni^2+^-affinity chromatography (Fig. 4a–c), and confirmed to be *Tgf2*TPase components by mass spectrometry analysis following trypsin digestion (Fig. 4d–f). The recombinant L-*Tgf2*TPase was previously obtained^27^.

**Figure 5:**
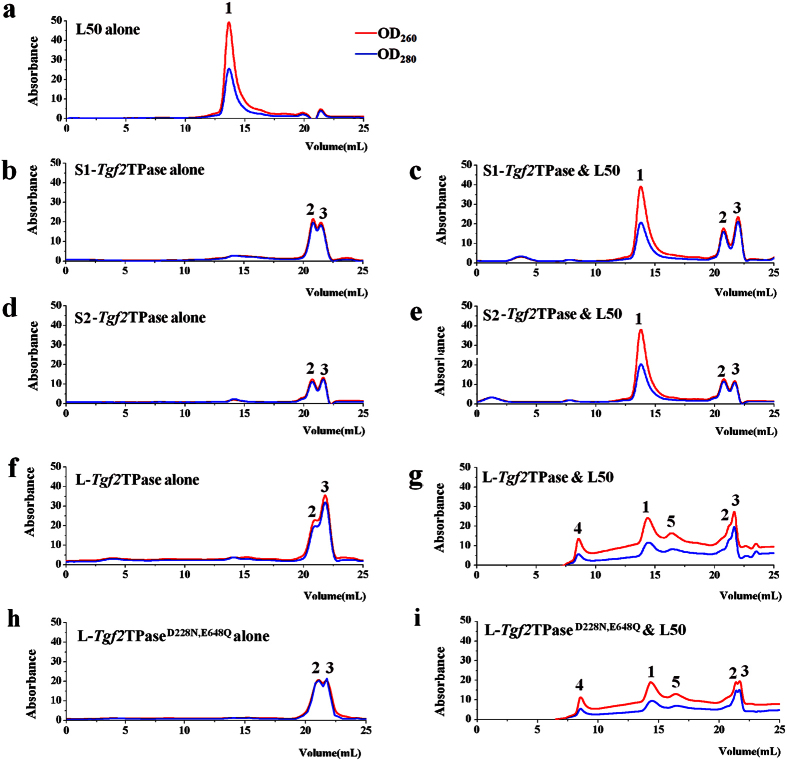
DNA-binding activity of S1-, S2- or L-*Tgf2*TPases analyzed by size exclusion chromatography. (**a**) Elution profiles of the L50 double-stranded DNA probe alone. (**b,c**) Elution profiles of S1-*Tgf2*TPase alone (**b**) or a mixture of S1-*Tgf2*TPase and L50 (**c**). (**d,e**) Elution profiles of S2-*Tgf2*TPase alone (**d**) or a mixture of S2-*Tgf2*TPase and L50 (**e**). (**f,g**) Elution profiles of L-*Tgf2*TPase alone (**f**) or a mixture of L-*Tgf2*TPase and L50 (**g**). (**h,i**) Elution profiles of L-*Tgf2*TPase^D228N, E648Q^ alone (**h**) or a mixture of L-*Tgf2*TPase^D228N, E648Q^ and L50 (**i**). Based on elution times, peak 1 corresponds to the L50 probe and peaks 2 and 3 represent *Tgf2*TPase dimers and monomers, respectively. Peaks 4 and 5 are new multimeric complexes formed from presumptive binding of the L50 probe and the L-*Tgf2*TPase.

For evaluation of DNA-binding activity *in vitro*, the size exclusion chromatography elution profiles of *Tgf2*TPases were investigated. As shown in Fig. 5, S1-, S2-, L-*Tgf2*TPase and L-*Tgf2*TPase^D228N, E648Q^ displayed characteristic protein profiles of monomer (peak 3) and dimer (peak 2) prior to DNA binding (Fig. 5b,d,f,h), with OD_260_ and OD_280_ being approximately equal. In contrast, the L50, double-stranded DNA probe exhibited a nucleic acid profile, with OD_260_ higher than OD_280_ (peak 1 in Fig. 5a). When S1- or S2-*Tgf2*TPase and L50 were mixed at a 1:1 molar ratio of protein and eluted, no other complex peaks were found except for peaks 1, 2 and 3 (Fig. 5c,e), suggesting that S1- and S2-*Tgf2*TPase did not bind well with L50 to form DNA-*Tgf2*TPase complexes at the concentration ratio tested.

However, there was a marked change in the peak characteristics when the L-*Tgf2*TPase and L50 mixture was eluted (Fig. 5g). Two new peaks (4 and 5) were seen before the L-*Tgf2*TPase protein peak, accompanied by a decrease in the L50 probe peak 1 and L-*Tgf2*TPase peaks 2 and 3 (Fig. 5g), when compared with peak 1 in L50 alone (Fig. 5a) and peaks 2 and 3 in L-*Tgf2*TPase alone (Fig. 5f). Due to the formation of L-*Tgf2*TPase-DNA complexes, the baseline for the 260 trace was significantly raised above the 280 profile in Fig. 5g. Moreover, peak 4 eluted faster than the peak 5 and the DNA probe peak, which implied that L-*Tgf2*TPase likely interacted with the DNA probe in the form of oligomerization containing more than two protein molecules (Fig. 5g). Since the L-*Tgf2*TPase^D228N, E648Q^ recombinant protein has an intact N-terminal domain, like L-*Tgf2*TPase, peaks 4 and 5 were also found when L-*Tgf2*TPase^D228N, E648Q^ and L50 mixture was eluted (Fig. 5i). In the negative control mixture of S1-, S2-, L-*Tgf2*TPase or L-*Tgf2*TPase^D228N, E648Q^ with random 50-mer double-stranded DNA C50, the above mentioned changes were not seen (Fig. S1). These results suggest that the N-terminal zinc finger domain in *Tgf2* transposase is involved in binding of the transposase to *Tgf2* terminal sequences.

### Truncated Tgf2 transposases have decreased transgenic efficiency

**Figure 6:**
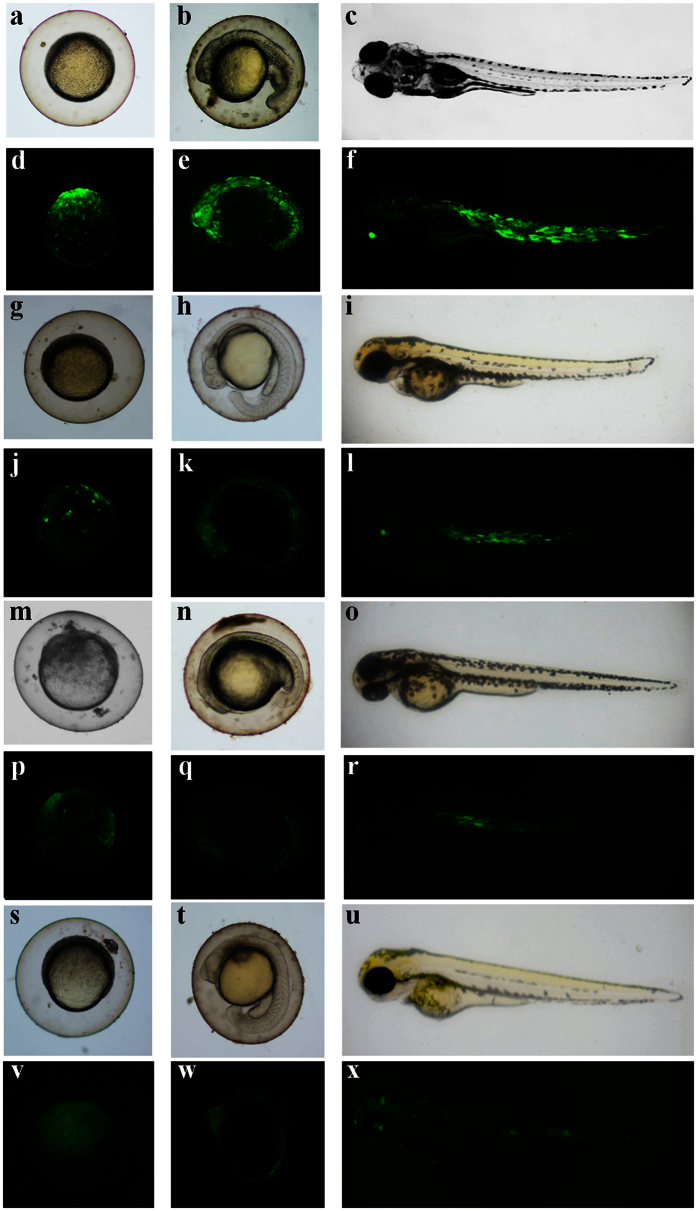
Microscopy images of EGFP expression in zebrafish embryos after microinjection of S1-, S2- or L-*Tgf2*TPase with donor construct pTgf2-EF1α-EGFP at the 1-2-cell stage. Light (**a**–**c**, **g**–**i**, **m**–**o**, **s**–**u**) and fluorescence (**d**–**f**, **j**–**l**, **p**–**r**, **v**–**x**) microscopy images of zebrafish embryos were shown at 12 hpf (**a**, **d**, **g**, **j**, **m**, **p**, **s**, and **v**), 24 hpf (**b, c, h, k, n, q, t,** and **w**) and 96 hpf (**c, f, i, l, o, r, u,** and **x**). Panels a–f are embryos treated with L-*Tgf*2TPase, panels g-l were treated with S1-*Tgf*2TPase, panels m-r were treated with S2-*Tgf*2TPase and panels s-x injected with donor plasmid pTgf2-EF1α-EGFP alone as controls.

To determine if the N-terminal zinc finger domain in *Tgf2* transposase has any effect on the transgenic efficiency during DNA transposition, we performed microinjection of *Tgf2*TPases into zebrafish embryos at the 1–2 cell stage. When 50 pg pTgf2-EF1α-EGFP was coinjected with 50 pg recombinant L-*Tgf2*TPase protein, an average of 68% of embryos showed strong and almost ubiquitous expression of EGFP (Table 1, Fig. 6d–f). EGFP fluorescence rates in embryos coinjected with recombinant S1- and S2-*Tgf2*TPase were reduced to 43% and 29% respectively (Table 1), with a weak expression of EGFP (Fig. 6j–l, p-r). In control embryos injected with donor plasmid alone or donor plasmid coinjected with recombinant L-*Tgf2*TPase^D228N, E648Q^, 24% and 22% of embryos showed mosaic EGFP expression (Table 1); the fluorescence in most of embryos should result from a weak expression of EGFP from the donor plasmid (Fig. 6v–x). PCR analysis of the transposition rate of EGFP in 3 month old adult zebrafish was performed as previously described^19^,^27^. The integration rate of EGFP when coinjected with L-*Tgf*2TPase reached 56% (Table 1). In comparison, significantly decreased integration rates were detected in zebrafish coinjected with S1- (21%) and S2-*Tgf2*TPases (25%, *p* < 0.01, Table 1). The integration rate of the EGFP sequence in control embryos injected with donor plasmid alone or coinjected with recombinant L-*Tgf2*TPase^D228N, E648Q^ was 8% and 7% respectively (Table 1).

### Truncated Tgf2 transposases exhibit altered TE excision and integration

**Figure 7:**
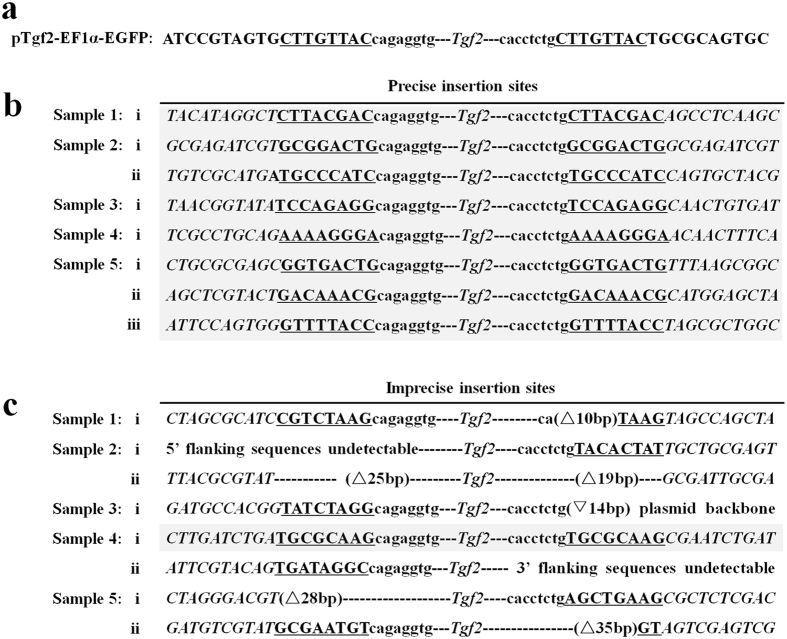
Exogenous *Tgf2* end regions and the surrounding insertion site sequences in the genome of 3 month old adult zebrafish. (**a**) Sequences of *Tgf2* end regions in the donor construct pTgf2-EF1α-EGFP. (**b**) Insertion sequences from 5 of 78 individuals listed in Table 2, with precise insertion sites after coinjection with pTgf2-EF1α-EGFP and L-*Tgf2*TPase. (**c**) Insertion sequences from all 5 samples in Table 2 with imprecise insertion sites after coinjection with pTgf2-EF1α-EGFP and L-*Tgf2*TPase. Precise insertion copies are shown with gray background. Lower case letters indicate the *Tgf2* end sequences. The 8 bp direct repeat signature of the target DNA associated with transposition is underscored. The surrounding sequences of the insertion sites are in italic. Triangles and inverted triangles represent deletions and insertions, respectively.

We further cloned the junctions of the integrated *Tgf2* element and the surrounding genomic DNA using inverse PCR. All 83, 21, 15, 4 and 5 EGFP-transgenic zebrafish adults that survived from each transgenic group (L-, S1-, S2-*Tgf2*TPase, L-*Tgf2*TPase^D228N, E648Q^ and control) respectively were examined (Table 1). A total of 143 insertion sites were identified from 83 zebrafish coinjected with L-*Tgf2*TPase (Table 2). There were 1 to 3 genomic integration sites in the genome of the zebrafish, and the average copy number was 1.7 (143/83). Most of L-*Tgf2*TPase injected fish (95%, Table 2) demonstrated intact TE end integration, indicating accurate excision and insertion during transposition and creation of complete 8 bp TSD signatures adjacent to both ends of *Tgf2* at the insertion sites (Fig. 7b). Among the 83 EGFP-transgenic positive zebrafish, 78 individuals (94%) had accurate insertions (Table 2). The remaining 6% (5/83) had partial deletion of transposon ends and/or plasmid backbone insertion/deletion, as well as incomplete 8 bp TSDs (Table 2; Fig. 7c), indicating imprecise excisions and insertions have occurred during transposition. In contrast, only 14% of individuals (3/21) with the S1-*Tgf2*TPase injections had precise transposition, while 67% of individuals (14/21) demonstrated imprecise integration, similar to the pattern in Fig. 7b,c. The remaining 19% (4/21) did not have any detectable insertion, which may be due to the absence of the primer binding region during splinkerette PCR. In all the insertion sites detected, the precise insertion rate was only 21% (4/21, Table 2). Consistently, only 13% (2/15) of individuals with the S2-*Tgf2*TPase injections exhibited precise genomic integration, while 67% (10/15) of individuals demonstrated imprecise insertion, similar to the pattern in Fig. 7b,c; the remaining 20% (3/15) had no detectable insertion. In all insertion sites detected with S2-*Tgf2*TPase injections, the precise insertion rate was only 20% (3/15, Table 2). The flanking sequences of the transposon insertion sites in 4 individuals coinjected with recombinant L-*Tgf2*TPase^D228N, E648Q^ were not detected by splinkerette PCR. Moreover, only 1 of 5 individuals from control embryos injected with donor plasmid alone had imprecise insertion (Table 2).

EGFP-transgenic fish were then raised to maturity and crossed with wild type zebrafish, and EGFP fluorescence expression in F1 embryos was examined at 24 hpf, to determine the existence of germline transmission^37^. Among genomic precise integration groups of L-, S1-, and S2-*Tgf2*TPase, 91% (71/78), 67% (2/3), and 100% (2/2) founders were able to transmit EGFP to their F1 embryos (Table 2); EGFP fluorescence expression in F1 embryo ranged from 20% to 78% (data not shown). Although the ratio of precise transposition in S1- or S2-*Tgf2*TPase populations was significantly reduced, EGFP-transgenic individuals with precise integration could efficiently transmit EGFP to their offspring. Taken together, our results indicate that truncated *Tgf2* transposases not only severely impair their *in vivo* transgenic efficiency, but also negatively impact on precision of DNA transposition.

## Discussion

In this study, the domain architecture of full-length *Tgf2* transposase was predicted (from N- to C-terminus) based on bioinformatics analysis. Four domains were identified: (1) an N-terminal zinc finger domain, that presumably coordinates Zn^2+^ through a conserved Cys_2_-His_2_ motif, and participates in binding to DNA^38^; (2) a dimerization domain^13^,^24^,^29^,^39^; (3) a C-terminus RNase-H domain that is a critical domain for DNA cleavage and integration^15^,^33^; and (4) a monopartite nuclear localization signal (NLS) found in the C-terminus^25^. These functional domains of *Tgf2* transposase are consistent with the analysis of conserved amino acids from *hAT* transposases^34^. Despite very limited sequence similarity among these *hAT* transposases, it seems likely that they share common mechanistic and structural features^24^,^34^. Since only a few *hAT* transposases have been studied in detail^24^, our successful purification of N-terminal truncated recombinant transposases makes it possible to experimentally verify the functional domains of the *Tgf2* transposase.

The BED zinc finger domain was initially described as Cx_2_Cx_n_Hx_3–5_(H/C) and have been proposed to bind DNA and to coordinate Zn^2+^ through a conserved CCHH or CCHC motif^38^. The zinc finger domain at residues 65–120 of the *Tgf2* transposase has the structure Cx_2_Cx_19_Hx_4_H. L-*Tgf2*TPase and L-*Tgf2*TPase^D228N, E648Q^ with intact zinc finger domains can bind to the L50 DNA probe, which consists of the 11-bp terminal inverted repeat (TIR) and five subterminal repeats (STRs) from left end of the *Tgf2* element (Fig. S2). In contrast, S1- and S2-*Tgf2*TPase do not bind well to probe, as suggested by size exclusion chromatography analysis. Accumulating data indicate that *hAT* transposases recognize their transposon tips in a bipartite manner, with weaker transposase binding to the TIRs and stronger binding by an N-terminal domain to these STRs^24^,^34^,^41^,^43^. For the *Ac* transposase, the zinc finger domain (Cx_4_Cx_17_Hx_4_H) is split into two subdomains, in which the C-terminal subdomain (Cx_4_C) appeared essential for binding to both sequences, while the N-terminal half (Hx_4_H) appears to bind to the TIRs but not to the STRs^40^,^41^. These data suggest that the zinc finger of *hAT* transposases is capable of binding to the TIRs and the STRs within the TE ends. Considering that S1- and S2-*Tgf2*TPases are distinguishable from L-*Tgf2*TPase only in the N-terminal region, this loss of DNA binding activity seen is likely due to the lack of the zinc finger domain.

The repeated subterminal repeats are haphazardly present within both ends of *hAT* transposons and are a defining feature of this superfamily^24^,^41^,^42^. The *Tgf2* elements are found to include 17 STR copies in the L end and 18 copies in the R end (Fig. S2). The zinc finger domain interacts with the outermost subterminal repeat on each end and this is important for both cleavage and strand transfer^41^,^43^. In the present study, L-*Tgf2*TPase-mediated gene transfer in the adult zebrafish genome *in vivo* occurred at a significantly higher efficiency than that mediated by zinc finger domain truncated *Tgf2*TPases (*p* < 0.01). In agreement with our results, the N-terminal deletion *IS911, Himar1* and *Sleeping Beauty* transposases containing the catalytic domain also showed downregulation of transposition activity^44^,^45^,^46^. In comparison with truncated *Tgf2*TPase, L-*Tgf2*TPase could accurately catalyze gene excision at TE ends and integration at zebrafish genome with complete TSD signatures adjacent to both ends of *Tgf2* at the insertion sites. These results indicate that both transposition efficiency and accuracy of the *Tgf2* system depends on zinc finger domain. The zinc finger domain interaction with both TIRs and STRs within the transposon ends could improve insertion fidelity, but the underlying mechanism is still an area of active investigation^24^,^34^.

During TE transposition, transposases tend to form transpososomes that contain multiple transposase monomers^24^,^39^. The *hAT* superfamily transposase *Hermes* can generate an unusually ring-shaped octamer *in vivo*, on the basis of crystal structure and negative staining electron microscopy analysis^13^,^24^. The *Tc1*/*mariner Mos1* and *Sleeping Beauty* transposases can also generate oligomers containing more than two molecules^5^,^28^. Due to the presence of a dimerization domain between aa 153 and 213, our data indicate that the S1-, S2- and L-*Tgf2*TPase can form dimers in solution prior to DNA binding, and sequential multimerization occurs concomitant with L-*Tgf2*TPase-DNA complex formation. The multimeric complexes contain multiple specific DNA-binding domains. The avidity provided by multiple sites of interaction could allow a transposase to locate its transposon ends amidst a sea of chromosomal DNA^24^. In addition to mediating the formation of dimers, the other function of dimerization domain in *hAT* transposases is to perform a weak DNA-binding^34^. Side chains from three amino acids (R107, F109, and S110) within dimerization domain interact with the sugar phosphate backbone of the *Hermes* L TIR between bp 6 and 8^24^. Moreover, the α-helices from insertion domain also bind to the *Hermes* transposon DNA^24^,^34^. These alternative DNA-binding motifs within the C-terminal dimerization and insertion domains are also conserved in *Tgf2* transposase. Since the alternative binding is relatively weak, the binding peaks are undetectable when the truncated *Tgf2*TPase and L50 mixture was eluted in our *in vitro* DNA-binding activity assay. The additional DNA binding sequences help to explain why truncated *Tgf2*TPases lost the zinc finger domain still have a moderate TE integration rate, although integration occurs imprecisely.

In summary, we predicted the functional domains in the full length goldfish *Tgf2* transposase by *in silico* analysis. The N-terminal zinc finger domain of *Tgf2* transposase was found to be responsible for the DNA-binding activity towards specific *Tgf2* end sequences, which had consequent effects on the gene-transfer efficiency. This DNA-binding domain is essential for mediating accurate excision and integration of the *Tgf2* element during *in vivo* transposition. The EGFP transgenic individuals with precise integration could efficiently transmit EGFP to their offspring, indicating germline transmission have occurred during transposition. Furthermore, our results demonstrate that D228N and E648Q mutations lead to knock out of *Tgf2* transposase function, which gives the experimental support that the proposed DDE motif forms the active center in the *Tgf2* transposase. Our efforts in elucidating the structure of *Tgf2* transposase provide insights into the transposition process and suggest application to further scientific investigations.

## Additional Information

**How to cite this article**: Jiang, X.-Y. *et al*. The N-terminal zinc finger domain of *Tgf2* transposase contributes to DNA binding and to transposition activity. *Sci. Rep.* **6**, 27101; doi: 10.1038/srep27101 (2016).

## Supplementary Material

Supplementary Information

## Figures and Tables

**Table 1:** Transposition efficiencies of N-terminal truncated *Tgf2*TPases in zebrafish.

Injection batches	No. of embryos			No. of adults		
	Examined	EGFP- expressed	Expressed rate (%)	Survived	EGFP- insertion	Insertion rate (%)
pTgf2-EF1α-EGFP + S1-*Tgf2*TPase	88	32	36	30	7	23
	130	70	58	64	10	16
	46	19	41	16	4	25
Average	–	–	43	–	–	21^b^
pTgf2-EF1α-EGFP + S2-*Tgf2*TPase	70	17	24	17	4	23
	110	37	34	35	7	20
	45	13	29	13	4	31
Average	–	–	29	–	–	25^b^
pTgf2-EF1α-EGFP + L-*Tgf2*TPase	90	60	67	55	28	51
	90	62	69	60	36	60
	56	38	68	34	19	56
Average	–	–	68	–	–	56^c^
pTgf2-EF1α-EGFP + L-*Tgf2*TPase^D228N, E648Q^	85	20	23	18	2	11
	93	21	23	19	1	5
	75	16	21	14	1	7
Average	–	–	22	–	–	7^a^
Control	86	21	24	18	2	11
	95	25	26	22	1	4
	86	20	23	18	2	11
Average	–	–	24	–	–	8^a^

Control is injected pTgf2-EF1α-EGFP plasmid only. Different letters is significant *p* < 0.01.

**Table 2:** Insertion accuracy and germline transmission of N-terminal truncated *Tgf2*TPases in EGFP-transgenic adult zebrafish at 3-month age.

Injection batches	No. of fish examined^a^	No. of fish with precise insertion sites	No. of fish with imprecise insertion sites	No. of fish with undetectable insertion sites	Total precise insertion sites	Total imprecise insertion sites	Precise insertion sites/total insertion sites (%)
pTgf2-EF1α-EGFP + S1-*Tgf2*TPase	21	3 (2)	14 (4)	4 (1)	4	15	21
pTgf2-EF1α-EGFP + S2-*Tgf2*TPase	15	2 (2)	10 (4)	3 (0)	3	12	20
pTgf2-EF1α-EGFP + L-*Tgf2*TPase	83	78 (71)	5 (2)	0	136	7	95**
pTgf2-EF1α-EGFP + L-*Tgf2*TPase^D228N, E648Q^	4	0	0	4 (1)	0	0	0
Control	5	0	1 (1)	4 (0)	0	1	0

^a^The EGFP-transgenic zebrafish from Table 1 were examined by splinkerette PCR. The number of fish demonstrating germline transmission is indicated in parentheses. Control was injected with pTgf2-EF1α-EGFP plasmid only. **p < 0.01 versus control.
