# Supplementary material for: The N-terminal zinc finger domain of Tgf2 transposase contributes to DNA binding and to transposition activity
Source: Sci Rep. 2016 Jun 2;6:27101. doi: 10.1038/srep27101 (PMC4890040; doi:10.1038/srep27101)
Supplement: Supplementary Information [file srep27101-s1.pdf]

The N-terminal zinc finger domain of *Tgf2* transposase contributes to DNA binding and to transposition activity

Xia-Yun Jiang[1, 2a], Fei Hou[1a], Xiao-Dan Shen[2], Xue-Di Du[3], Hai-Li Xu[1] and Shu-Ming Zou[2*]

1 College of Food Science and Technology, Shanghai Ocean University, Huchenghuan Road 999, Shanghai 201306, China

2 Key Laboratory of Freshwater Aquatic Genetic Resources, Shanghai Ocean University, Huchenghuan Road 999, Shanghai 201306, China

3 College of animal science and technology, Yangzhou University, Wenhui Road 48, Yangzhou 225009, China

[a] These authors contributed equally to this work

* To whom correspondence should be addressed.

E-mail: smzou@shou.edu.cn

SUPPLEMENTARY FIGURES

Supplemental Figure 1: Size exclusion chromatography analysis of S1-, S2- or L-*Tgf2*TPases binding with random 50-mer double stranded DNA control C50.

(a) Elution profiles of C50 double stranded DNA probe alone; (b-c) Elution profiles of S1-*Tgf2*TPase alone (b) or a mixture of S1-*Tgf2*TPase and C50 (c); (d-e) Elution profiles of S2-*Tgf2*TPase alone (d) or a mixture of S2-*Tgf2*TPase and C50 (e). (f-g) Elution profiles of L-*Tgf2*TPase alone (f) or a mixture of L-*Tgf2*TPase and C50 (g). (h-i) Elution profiles of L-*Tgf2*TPase$^{D228N, E648Q}$ alone (h) or a mixture of L-*Tgf2*TPase$^{D228N, E648Q}$ and C50 (i). Based on elution times, peak 1 corresponds to C50 probes and peaks 2 and 3 represent *Tgf2*TPase dimers and monomers, respectively.

Supplemental Figure 2: Terminal inverted repeats (TIRs) and subterminal repeats (STRs) within the left (220bp) and right (185bp) ends of the *Tgf2* element. The 11 bp TIRs are underscored. Arrows suggest the occurrence of A/GAGTA STR motifs. Gray sequences show double-stranded DNA L50 probe for size exclusion chromatography.

Supplemental Figure 1:

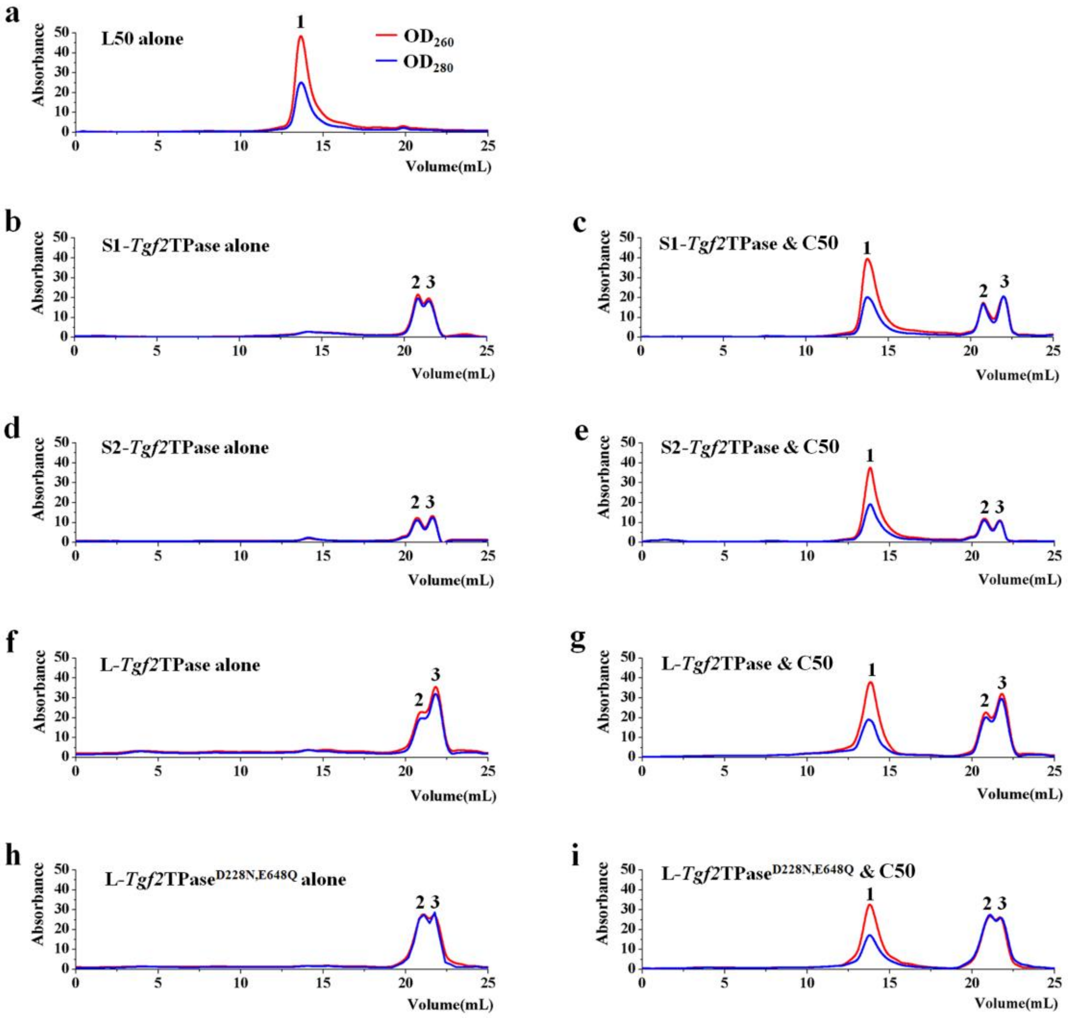

Supplemental Figure 2:

***Tgf2* left-end**

```
         10        20        30        40        50        60        70
CAGAGGTGTAAAAGTACTTGAGTAATTTTACTTGATTACTGTACTTAAGTATTATTTTTGGGGATTTTTA
GTCTCCACATTTTCATGAACTCATTAAAATGAACTAATGACATGAATTCATAATAAAAACCCCTAAAAAT

         80        90       100       110       120       130       140
CTTTACTTGAGTACAATTAAAAATCAATACTTTTACTTTTACTTAATTACATTTTTTTTAGAAAAAAAGT
GAAATGAACTCATGTTAATTTTTAGTTATGAAAATGAAAATGAATTAATGTAAAAAAAATCTTTTTTTCA

        150       160       170       180       190       200       210
ACTTTTTACTCCTTACAATTTTATTTACAGTCAAAAAGTACTTATTTTTTGGAGATCACTTCATTCTATT
TGAAAAATGAGGAATGTTAAAATAAATGTCAGTTTTTCATGAATAAAAAACCTCTAGTGAAGTAAGATAA

        220                4540      4550      4560      4570      4580
TTCCCTTGCT .............. TCTAGTTTTCATTCTTGCTTTTACTTTTACTTCTTTAATACTCAA
AAGGGAACGA .............. AGATCAAAAGTAAGAACGAAAATGAAAATGAAGAAATTATGAGTT

       4590      4600      4610      4620      4630      4640      4650
GTACAATTTTAATGGAGTACTTTTTTACTTTTACTCAAGTAAGATTCTAGCCAGATACTTTTACTTTTAA
CATGTTAAAATTACCTCATGAAAAAATGAAAATGAGTTCATTCTAAGATCGGTCTATGAAAATGAAAATT

       4660      4670      4680      4690      4700      4710      4720
TTGAGTAAAATTTTCCCTAAGTACTTGTACTTTCACTTGAGTAAAATTTTTGAGTACTTTTACACCTCTG
AACTCATTTTAAAAGGGATTCATGAACATGAAAGTGAACTCATTTTAAAAACTCATGAAAATGTGGAGAC
```

***Tgf2* right-end**
